# Parental self-efficacy managing a child’s medications and treatments: adaptation of a PROMIS measure

**DOI:** 10.1186/s41687-023-00549-z

**Published:** 2023-02-03

**Authors:** Carolyn C. Foster, Courtney K. Blackwell, Kristin Kan, Luis Morales, David Cella, Sara Shaunfield

**Affiliations:** 1 Division of Advanced General Pediatrics and Primary Care, Department of Pediatrics, Northwestern University Feinberg School of Medicine, Chicago, IL USA; 2 Mary Ann & J. Milburn Smith Child Health Outcomes, Research, and Evaluation Center, Stanley Manne Children’s Research Institute, Ann & Robert H. Lurie Children’s Hospital of Chicago, 225 East Chicago Avenue, Box 162, Chicago, IL 60611 USA; 3 Depatment of Medical Social Sciences, Northwestern University Feinberg School of Medicine, Chicago, IL USA

**Keywords:** Self-efficacy, Patient-reported outcome measure, Children with special health care needs, Children with medical complexity, Children with disability

## Abstract

**Purpose:**

Self-efficacy is important for managing chronic conditions; however, its measurement in pediatric healthcare settings remains rare. The goal of this project was to adapt an existing disease-agnostic adult self-efficacy patient reported outcome (PRO) measure to enhance suitability of items for measuring the self-efficacy of parents that manage their children’s health conditions.

**Methods:**

We adapted the existing Patient-Reported Outcomes Measurement Information System® (PROMIS®) adult self-efficacy healthcare measure to parental voice. First, a targeted literature review informed rephrasing of the adult items and identification of new pediatric-specific content. The initial item pool was revised based on input from 12 multidisciplinary experts. Next cognitive interviews of adapted items were simultaneously conducted with English and Spanish-speaking parents of pediatric patients with a range of chronic and/or disabling conditions recruited from a Midwestern children’s hospital to finalize the measure.

**Results:**

Findings resulted in an initial item pool of 33 pediatric-specific items which were narrowed to 31 draft items based on expert input. Parent cognitive interview findings (N = 26) informed further item reduction resulting in a final measure consisting of 30 items representing nine domains. Fourteen items are relevant to children regardless of condition severity (e.g., health care information/decision making; symptom identification/management) and 16 items are relevant to children with specific health care needs (e.g., medication usage, equipment).

**Conclusion:**

We conducted a first step in developing a condition-agnostic, PRO measure of parental self-efficacy managing their children’s chronic and/or disabling conditions that is acceptable and understandable to English and Spanish-speaking parents.

**Supplementary Information:**

The online version contains supplementary material available at 10.1186/s41687-023-00549-z.

## Plain English Summary

Self-efficacy, which is someone’s confidence in completing a task, is important for managing a chronic health condition. Knowing parents’ self-efficacy managing their children’s health conditions may be an important step in supporting their children’s health but no single measure is available for diverse sets of conditions. In this paper, we present the development of a new patient reported outcomes measure designed to assess self-efficacy of parents managing their child’s chronic and/or disabling conditions. We found that the measure is both acceptable and understandable to English and Spanish speakers and may be useful to proactively identify parents in need of additional supports at hospital discharge or at the time of a new diagnosis.

**Supplementary Information:**

The online version contains supplementary material available at 10.1186/s41687-023-00549-z.

## Introduction

After a child’s visit to a pediatrician’s office or discharge from a hospital, providers typically instruct family caregivers (hereafter *parents*), and sometimes the children themselves, to complete a set of health care tasks to ensure the child’s health going forward. These tasks may range from administering a child’s medication, to scheduling a child’s follow-up appointment, to observing a child for a change in symptoms. Parent confidence in their ability to do these tasks on behalf of their child is a form of self-efficacy, long understood to be an important component of the management of chronic conditions [1].

Self-efficacy is defined as “confidence in one’s ability to exert control over one’s own motivation and behavior regardless of the outcome,” (pages 2513–2514) [2]. Research in adults and children demonstrates that higher self-efficacy is associated with more adaptive coping strategies subsequently linked to improved health outcomes [2–7]. Notably, self-efficacy is not necessarily a measurement of skill or ability, but a reflection of the confidence a person has of their ability to perform a task and has been shown to play a key role in task execution [1]. Extant research indicates that supporting a parent’s self-efficacy is a critical step to ensuring the successful execution of a child’s home health care regimen in chronic childhood conditions [3–8].

Patient-reported outcome (PRO) measures, such as those used in these aforementioned studies, are brief, standardized, and evidence-based survey-based tools that provide data about patient symptoms, quality-of-life, and access to care [9–11]. PRO measures have been successfully developed for parent proxy report of child health, including for those with specific disabilities and chronic diseases [9, 10, 12–17]. PRO measures already exist to capture parental activation generally [18] or self-efficacy in managing specific pediatric conditions [3–5]. However, to our knowledge there is no freely available condition-agnostic measure of parent self-efficacy managing a child’s medical care. Similarly, there are no self-efficacy measure that address medical tasks relevant to children with medical complexity (CMC) who have disability and/or technology dependence. Given that children often rely on caregivers to perform medical tasks due to their developmental ability, either because of age or condition-related impairment [8], a need exists for a validated, condition-agnostic, self-efficacy PRO measure to assess the confidence of parents managing their children’s chronic and even complex and/or disabling conditions. While a parent’s self-efficacy may be primarily an intermediate result in a series of steps towards ensuring a health outcome for the child, a parent’s low or high self-efficacy can itself be a measurable outcome following education and training interventions.

The National Institutes of Health funded Patient-Reported Outcomes Measurement Information System® (PROMIS®) has developed standardized measures to evaluate and monitor physical, mental, and social health in adults and children [19, 20]. These include a PROMIS® self-efficacy PRO measure that assesses adults’ self-efficacy in managing their own medications and treatments [21, 22]. Consistent with the PROMIS approach, we define a PRO as any report of a symptom, perception, or experience that is best reported by the patient or patient proxy [20]. Therefore, this project conceptualizes parental self-efficacy as a measurable PRO about the confidence experienced by a patient’s parent. The goal of this project was to adapt the adult self-efficacy measure to the pediatric context to facilitate measurement of self-efficacy in parents whose children have a range of chronic health conditions, including parents of CMC. The study is a first step in developing content validation for a self-efficacy measure that can be used to assess the level of parental self-efficacy at discrete points in care.

## Methods

The Ann & Robert H. Lurie Children’s Hospital of Chicago’s Institutional Review Board approved all study procedures. Signed consent was obtained for parental participation with consent to publish results. Cross-sectional parental survey data were collected and managed using REDCap (Research Electronic Data Capture) tools hosted at Northwestern University [23].

### Phase 1: Initial PRO item adaptation

**Fig. 1:**
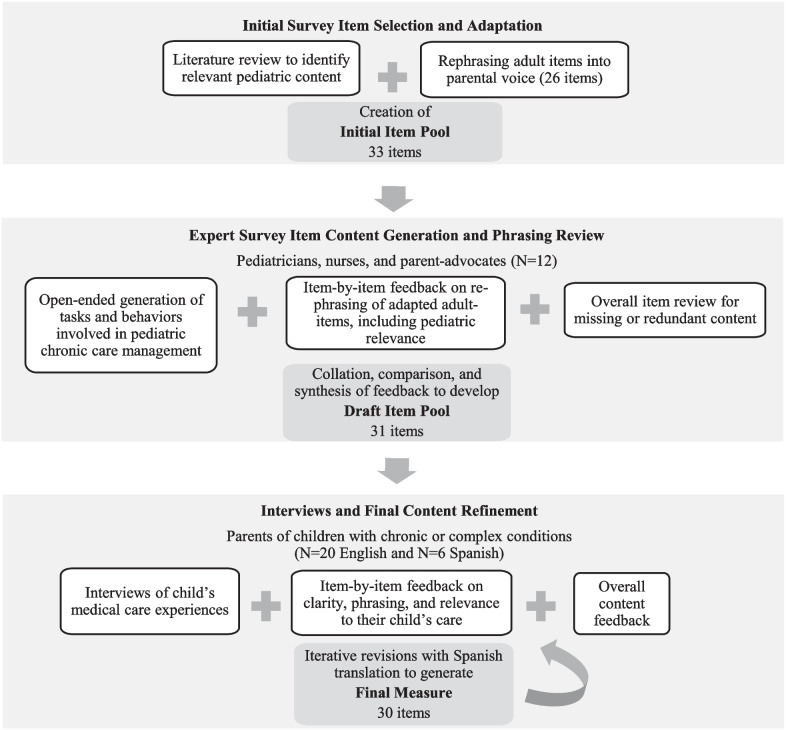
Methods for development of a survey measure of parental self-efficacy to manage a child’s medications and treatments

The methods for adapting the PROMIS adult self-efficacy measure to parent-reported self-efficacy is show in Fig. 1. We followed established methods to ensure the content validity of the adapted items in a pediatric chronic disease and medical complexity context, and to ensure the questions were understandable to a diverse English and Spanish-speaking parent population [19, 24–29].

First, we conducted a *targeted literature review* to identify current measures of self-efficacy in management of chronic diseases in adults and children. The primary investigator (CF) used this review to generate new pediatric-focused content not captured in the PROMIS adult self-efficacy items. Seven newly drafted items were developed for relevance to children with a range of medical complexity, including those who need skilled medical tasks at home (e.g., medication administration by tube, suctioning, ventilator equipment) or assistance with activities of daily living (ADLs) due to developmental delay or disability (e.g., transfers, diapering). The 26 items of the PROMIS adult self-efficacy measure were rephrased into parent voice (CF). For example, if the original adult item read, “I can take several medications on different schedules,” the adapted parent item was, “I can give my child several medications on different schedules.” Consistent with PROMIS methods, phrasing targeted a 6th grade reading level [27]. Together, the proposed pediatric-focused content and adapted items were combined to create an *Initial Item Pool* (33 items).

### Phase 2: Expert PRO item content generation and phrasing review

Next, the *Initial Item Pool* was refined with input from a national multidisciplinary group of experts (N = 12) in the care of children with chronic disease and medical complexity. The experts were selected from leaders in pediatric care that span a spectrum of settings (i.e., inpatient, outpatient, home). Professional parent experts were identified from a national family advocacy organization focused on child health.

Each expert was given an open-ended prompt to list behaviors and tasks parents of children with chronic conditions would engage in to care for their child’s conditions, including CMC (e.g., make appointment, give medications) (Additional file 1: Appendix A). Experts were asked to rank the tasks in order of importance across a group of children with different medical conditions (0 = Not at all important to 5 = Extremely important). Then, each expert was given a table containing the *Initial Item Pool*, which included the original (adult) items (as applicable) alongside the proposed adapted parent items. Experts reviewed the table and were asked to: (a) indicate if the meaning of the pediatric version was clear, (b) propose re-wording if any, and (c) indicate if the item’s content reflected their experiences in the daily care of children with chronic and medically complex conditions. Finally, the experts were asked to review the PRO items together as a group and were asked to identify any missing or redundant content.

The research team then collated the expert generated content and organized it into categories (e.g., healthcare navigation, symptom management, decision-making). The expert generated content was compared to the *Initial Item Pool* to identify any new content, focusing on those categories with rankings of ≥ 4. Similarly, item-by-item feedback on rephrasing was collated and compared (CF, LM) using the established method of PROMIS item selection [27]. If a new phrase was recommended by more than one expert, new phrasing was chosen based on the most representative feedback of the group using a discussion-based process. Items considered by most experts to be redundant or irrelevant to pediatric care were removed. In addition to the discrete item feedback, thematic feedback was reviewed and reconciled about the caregiver’s self-efficacy concerns, attitudes, and beliefs, which informed development of new items or item revisions. Together, the new item content (7 items), adapted items (24), and overall feedback were synthesized to create the new *Draft Item Pool* (31 items).

### Phase 3: Parent cognitive interviews and final content refinement

The *Draft Item Pool* was further refined to clarify response options and wording using iterative combined concept elicitation and cognitive interviews of parents of children with chronic disease and medical complexity [19, 25–29]. First, patients were identified from a Midwestern independent children’s hospital electronic health data using the Pediatric Medical Complexity Algorithm (PMCA) [30]. Patients were included if less than 21 years of age, as this is recognized in the United States at the end of adolescent development. PMCA is a validated algorithm that distinguishes between groups of children with chronic health conditions using expert consensus definitions developed by the Centers of Excellence on Quality of Care Measures for Children with Complex Needs Working Group [30]. This national working group defines children with noncomplex chronic disease as those with chronic conditions expected to last at least 1 year and are commonly lifelong but can be episodic (e.g., type 1 diabetes, asthma). CMC are defined as having a chronic complex disease that had 2 or more significant chronic conditions, a progressive condition, need for continuous dependence on technology for at least 6 months, or active malignancy impacting life function; examples include spastic quadriplegic cerebral palsy, developmental delay, and chronic pulmonary disease.

After identifying patients who were either children with a noncomplex chronic condition or CMC, we approached their parent/legal guardian by phone and/or email to confirm that they were eligible for the interview. Eligibility criteria were: age ≥ 18, legal guardian (either biological or non-biological parent), and English or Spanish-speaking. Participants were consented prior to the start of the interview and given a $40 gift card after completing the study. Spanish and English-speaking participants were recruited and engaged simultaneously. All interviews were conducted using a secure remote video-conferencing software by a bilingual research coordinator (LM) using a semi-structured interview guide (Additional file 2: Appendix B); detailed field notes were taken. The cognitive interview guide was based on established procedures and was designed to elicit general feedback on instruction clarity, response options, format, and item comprehension [31, 32].

During each interview, parents were asked to list behaviors or tasks that related to managing their children’s medical condition(s) and rank the behaviors/tasks in order of importance. Then, parents were asked to complete the questionnaire containing the *Draft Item Pool* using an electronic link. Instructions on the draft questionnaire were the following, “Please respond to each question or statement by marking one box per row based on your current level of confidence…,” followed by the list of items. Response options were a 5-point graded scale (1 = “I am not confident at all” to 5 = “I am very confident”).

The interviewer asked a series of questions for each item: (a) what they thought about when answering the item, (b) how they would state the item in their own words (re-phrasing), (c) their confidence in responding (1 = very confident, 2 = confident, 3 = not at all confident), and (d) whether the item content was relevant to their child’s care (yes, no). Finally, parents were asked to consider the questions holistically to identify any missing or redundant content. Basic demographic information was also collected and analyzed with univariate statistics.

Iterative revisions with parents of both CMC and children with noncomplex chronic disease were then performed with Spanish translation of the items with parents with limited English proficiency whose preferred language was Spanish. Revised questions were then re-translated into English and tested again. Recruitment for this process was conducted in an iterative manner until no new feedback emerged [33] regarding item comprehensiveness, relevance, clarity, and comprehension to generate the *Final Measure* [27]. In addition to discrete item feedback, the thematic feedback was reviewed, reconciled, and summarized (CF, LM) in a manner that reflected parents’ concerns, attitudes, and beliefs about self-efficacy of managing their child’s health condition(s), noting any differences for CMC versus children with noncomplex chronic disease*.* Survey responses were analyzed using univariate statistics (Stata Statistical Software: Release 15. College Station, TX).

## Results

### Expert demographics and themes

Of the 12 expert participants, most were female (n = 10). Expert participants reflected a range of clinical experience, including pediatricians (n = 5) spanning general pediatrics, hospital-based medicine, home health care, pediatric physical medicine and rehabilitation, and developmental and behavioral pediatrics; pediatric nurses (n = 4) with experience in care coordination or clinical care of children with medical technology dependence; and parents of CMC (n = 3) in parent leadership roles.

### Initial adaptation

**Fig. 2:**
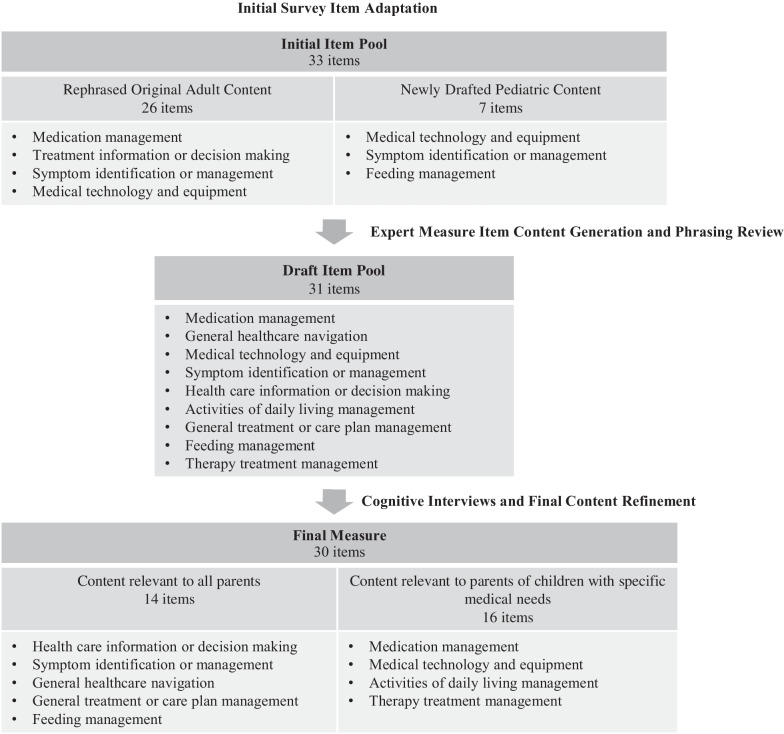
Results of development of a survey measure of parental self-efficacy to manage a child’s medications and treatments

Results of the process to adapt items from the existing adult PROMIS® self-efficacy PRO measure are shown in Fig. 2. All 26 items in the original adult measure were rephrased and 7 new items were drafted to capture content identified in the pediatric literature not covered by the original adult measure. New items were drafted to capture concepts of medical technology and equipment, symptoms, and feeding. Together, the adapted and newly drafted items resulted in an *Initial Item Pool* of 33 items.

### Expert input

Experts recommended a new item category reflecting parent’s confidence with healthcare navigation, including the use of telemedicine (Table 1). They also recommended moving away from the adult PRO’s focus on medication management and broadening the questions to include other forms of treatment. Specifically, support for activities of ADL and therapy services (e.g., physical therapy) were thought to be important parental care components relevant to a range of childhood conditions impacting development. Questions about medical technology and equipment were expanded to include not just correct use but also maintenance. The most frequently listed behaviors/tasks were medication administration and provider appointments. In summary, expert feedback led to the development of 16 new items, and modification of 12 draft items, and removal of 11 items, resulting a 31-item *Draft Item Pool* for parent evaluation.

**Table 1:** Concept elicitation for a family caregiver's self-efficacy managing their child's chronic health condition

Behavior or tasks	N
Give medications	20
Schedule and attend doctors appointments	10
Use child's health care equipment	9
Maintain and/or order child’s equipment and supplies	9
Know child’s health care plan	8
Support child’s therapy exercises	8
Communicate with health care team about child’s care, on as needed basis	7
Treat symptoms	7
Provide activities of daily living care, including safe transfers	7
Know when to seek care versus handle immediate medical emergency	6
Communicate using shared decision making around child's care	6
Identify changes in child's condition	6
Understand patient's diagnoses and health care plan	6
Give feeding regimen	4
Give non-medication treatment (e.g. vest)	4
Facilitate care at school or other location	4
Identify side-effects of medications or treatments	3
Obtain medications	3
Obtain medications	3
Facilitate care if parent is away	2
Other (e.g., wound care, safe home environment, health literacy, telehealth visit, access transportation, network, know resources, etc.)	1

Experts and parents are asked to list behaviors or tasks involved in managing or caring for a child’s medical condition(s)

The frequency the behavior/tasks was listed in general categories by frequency across the experts and interviewees

### Cognitive interviews

Parent cognitive interviews occurred between 11/2020–09/2021. Tables 2 and 3 detail the parent participant and child characteristics respectively. Twenty-six parents were interviewed of which two-thirds (n = 18) were parents of CMC with conditions such as cerebral palsy, organ transplant, and complex congenital heart disease. The others (n = 8) were parents of children with noncomplex chronic diseases, such as asthma, arthritis, and attention-deficient hyperactivity disorder. About one-third (n = 6) were Spanish speaking.

**Table 2:** Characteristics of interviewed parents

Characteristics	N (%)
*Age in years*	
Mean (SD)	42 (SD = 8.5)
*Gender identity*	
Male	2 (8)
Female	24 (92)
*Language interviewed conducted in*	
English	21 (81)
Spanish	5 (19)
*Ethnicity*	
Non-Hispanic/Latinx	19 (73)
Hispanic/Latinx	7 (27)
*Race*	
White	17 (65)
Black or African American	3 (12)
Asian	1 (3)
Other or not specified (left blank)	5 (19)
*Marital status*	
Married	16 (61)
Divorced or separated	5 (19)
Never Married	3 (12)
In a committed relationship	2 (8)
*Highest level of education*	
High school or equivalent	4 (15)
Some college, technical degree or associates degree	7 (27)
College degree (bachelor)	10 (39)
Advanced degree (masters or graduate)	5 (19)
*Current employment self-report*	
Stay-at-home parent	12 (46)
Full-time employed	8 (31)
Part-time employed	4 (15)
Unemployed or retired	2 (8)

N = 26

**Table 3:** Characteristics of interviewed parents’ children

Patient characteristics	N (%)
*Age*	
Mean (SD)	9 (SD = 6)
0–4 years	9 (35)
5–11 years	7 (27)
> 12 years	10 (38)
*Biological sex*	
Male	11 (42)
Female	14 (58)
*Payor*	
Public (medicaid)	15 (58)
Private (employer-based/commercial)	11 (42)
Conditions*	
Cerebral palsy (spastic diplegia or quadriplegia)	4 (15)
Prematurity with multiorgan sequalae	4 (15)
Genetic/metabolic syndrome with multiorgan involvement	4 (15)
Cancer	2 (8)
Organ transplant	2 (8)
Complex congenital heart disease	2 (8)
Muscular dystrophy	1 (4)
Simple congenital heart disease	1 (4)
Autism	1 (4)
Food allergy	1 (4)
Juvenile idiopathic arthritis	1 (4)
Asthma	1 (4)
Attention deficit and hyperactivity disorder	1 (4)
Epilepsy	1 (4)
Dermatomyositis	1 (4)
*Medical technology dependence (e.g. gastrostomy tube, ventilator, *etc*.)*	
Yes	13 (50)
No	13 (50)

N = 26

*Adds up to > 100% due to rounding and patient with more than one complex condition grouping

During the cognitive interviews, parents consistently reported that the response options were clear and appropriate. However, parents of CMC consistently highlighted concern with wording that did not specify the locus of control over an activity. Specifically, parents emphasized the importance of phrasing that focuses on what the parent has control over – for example, it is one thing to know the steps to contact their child’s healthcare team, and another to get ahold of their child’s health care team. Parents of CMC emphasized this also for phrasing around shared-decision making and communication, noting that just because they felt confident did not mean they always felt the healthcare team was receptive to their behaviors. Parents also recommended collecting information on how much time had passed since their children’s diagnoses to better interpret their responses; some reported that they had felt less confident early in their child’s diagnosis with some behaviors, but that their self-efficacy grew over time.

Parents of children with noncomplex chronic disease identified that some item content was not relevant to their children or care experiences including questions about medical technology or ADLs. Notably, this included medication because some parents reported that their child’s treatment only consisted of behavioral or therapeutic interventions. Also, a few parents of children with noncomplex chronic disease reported feeling unsure how to answer some of the questions for behaviors that their adolescent children had started taking responsibility for managing themselves. This led to additional instructions specifying that the measure intended to capture the parents’ self-efficacy regardless of their child’s behaviors.

Altogether, cognitive interview feedback altered phrasing in 7 items and 1 item was removed iteratively through the first n = 20 participants and confirmed with the final n = 6.

### Final questionnaire domains and items

The final questionnaire content (30 total items) representing nine domains can be found in Table 4. The content included domains ranging from general healthcare navigation to treatment planning. Fourteen items were relevant to all participants across domains related to healthcare information or decision making; symptom identification or management; general treatment management, general healthcare navigation, and feeding management. There were an additional 16 items that were relevant to children with specific healthcare needs: medication usage; medical technology or equipment; therapy treatment management; and ADL management.

**Table 4:** Survey items to assess family caregiver’s self-efficacy managing their child’s chronic health condition

Domain relevance	Domain	Item	Mean responses (SD)	Response range
All parents	Health care information or decision making	I know the healthcare condition(s) that affect my child	4.62 (0.61)	3–5
		I can work with my child’s doctor(s) to choose the treatment that seems right for my child, including the option of not giving any treatment	4.40 (0.87)	2–5
		I can actively participate in decisions about my child’s treatment	4.76 (0.5)	3–5
		I can find information to learn more about my child’s treatment	4.57 (0.7)	3–5
	Symptom identification or management	I know when my child needs to be seen by a healthcare provider, if sick	4.6 (0.70)	3–5
		I know what to do if my child has a medical emergency until help arrives	4.71 (0.55)	3–5
		I can figure out what my child needs when my child’s symptoms change	3.96 (0.87)	2–5
		I can tell when my child’s symptoms worsen	4.40 (1.0)	2–5
	General treatment management	I can follow my child’s full treatment plan (including medication, therapy, and other care)	4.57 (0.78)	2–5
		I know what to do if my child’s misses a medication or other type of treatment	4.38 (0.71)	3–5
	General healthcare navigation	I know the steps needed to get in contact with my child’s healthcare providers when I have a question or concern about my child’s care	4.54 (0.65)	3–5
		I know the steps needed to schedule my child’s healthcare appointments	4.73 (0.53)	4–5
		I know how to set up a telemedicine video visit for my child using an electronic device	4.00 (1.28)	1–5
	Feeding management	I can follow my child’s diet or feeding plan	4.83 (0.38)	4–5
Parents with children who have specific medical needs	Medication usage	I can continue my child’s medications or other treatment when we are away from home	4.58 (0.76)	2–5
		I know how to arrange for my child to receive medication or treatments at locations other than home, if needed	4.35 (0.98)	2–5
		I can tell the difference between when my child is having a medication side effect or experiencing symptoms of their condition(s)	4.04 (0.82)	2–5
		I can give my child’s medications when they are scheduled to be given	4.84 (0.37)	4–5
		I know how to give my child’s medications (such as by mouth or by tube)	4.79 (0.66)	2–5
		I know how to get my child’s medication refilled, if it is needed	4.71 (0.46)	4–5
		I can keep a list my child’s medication, including the medication doses and schedule	4.83 (0.48)	3–5
		I know what to do when my child’s medication refill seems different than usual	4.7 (0.64)	3–5
	Medical technology or equipment	I can use my child’s medical equipment by myself	4.47 (0.61)	3–5
		I can tell when parts of my child’s medical equipment, parts, or supplies needs to be replaced or repaired	4.44 (0.62)	3–5
		I can clean my child’s medical equipment	4.59 (0.62)	3–5
		I know the settings on my child’s medical equipment (such as a pump, monitor, ventilator, etc.)	4.56 (0.62)	3–5
		I can change my child’s disposable supplies or devices (such as diabetes pump, tracheostomy, line dressing, etc.)	4.50 (0.79)	3–5
		I can use my child’s mobility equipment, such as a wheelchair, walker, or lift	4.10 (1.3)	2–5
	Therapy treatment management	I can help my child do their therapy exercises	4.00 (1.0)	2–5
	Activities of daily living management	I can move my child safely	4.47 (0.68)	3–5

Parents are asked to, “Please respond to each question or statement by marking one box per row based on your CURRENT level of confidence…” with the response options of, “I am not confident at all (1), I am a little confident (2), I am somewhat confident (3), I am quite confident (4), I am very confident (5).” Please answer based on your comfort with these tasks, even if your child may do some of these themselves

Participants’ scored responses to the items are also shown in Table 4 (mean, standard deviation (SD) and range), with parents reporting a range of confidence across the items but with most respondents choosing response categories between 2 (little confident) or 3 (somewhat confident) to 5 (very confident). Mean item scores ranged from 3.96 (SD 0.87, range 1–5) regarding confidence with setting up a telemedicine video appointment to 4.83 (SD 0.37, range 4–5) and 4.83 (SD 0.38, range 4–5) for keeping a list of a child’s medications and following a child’s diet or feeding plan, respectively.

## Discussion

Despite the important role parent self-efficacy plays in the management of child health conditions at home on a day-to-day basis, to our knowledge, this adaptation has led to the first condition-agnostic, self-efficacy PRO measure to assess the confidence of parents managing their children’s chronic health conditions. In our this newly adapted Parental Measure of Self-Efficacy Managing a Child’s Medications and Treatments, we identified domains with items that had general applicability across children with noncomplex chronic conditions, and domains specific to those with more complex and/or disabling health conditions.

This PRO has the potential for a range of applications. First, we still know little about how a parent’s self-efficacy may change over time for children with complex conditions, particularly in cases where children require multiple subspecialists and medical equipment needs, nor do we know how self-efficacy may factor into their children’s acute healthcare (e.g., emergency room visits) or de-escalation of care. For example, children born prematurely often experience a range of health sequelae that can require parents to be prepared to provide care that involves feeding equipment, complex medication regimens, and multiple subspecialty follow-ups. Our previous research indicates, for example, that parents of children with chronic disease must not only be able to demonstrate their ability to place a tube in their child’s nose to support feeding, but have the confidence to do so, prior to a successful discharge home from the hospital [34].

Low parental self-efficacy for completing complex care tasks may be predictors of subsequent emergency department use and adversely impact parent quality of life [8], in addition to the impacts on the child’s health and development. Specifically, future work should test whether parents with lower self-efficacy in performing their children’s health care related tasks means their child is less likely to obtain their prescribed treatment regimen and thereby less likely to have enhanced health outcomes. Also, future work should test whether parents who have lower self-efficacy are more likely to seek emergency or other unplanned care services than those who are more confident with executing their children’s health care routine.

The newly developed Parental Measure of Self-Efficacy Managing a Child’s Medications and Treatments could be used to proactively identify parents in need of additional supports. Given participants’ insights that time since diagnosis may be a marker of confidence, this PRO may also be used in an outpatient setting for parents of children with a new, chronic diagnosis. Use of the PRO in this case may identify parents who have not yet had to interface heavily with the healthcare system and potentially flag opportunities to better support their behaviors and skills in managing their child’s newly diagnosed condition. To enable these potential applications, future work could examine the correlations between parent self-efficacy and: (1) post-discharge acute unplanned care use or (2) newly diagnosed patients’ engagement with their health care team.

Whereas the adult measure focused heavily on medication management as a health care task, our expert panel and parent interview participants outlined how a child’s treatment does not necessarily include medication management and that the self-efficacy measure tasks must be inclusive of non-pharmacologic treatment. This finding reflects a broad base of literature that highlights the importance of physical therapy, occupational therapy, speech/language pathology, and behavioral therapy to improve health outcomes in a range of pediatric conditions [35, 36]. Given that parent participation in therapy services are important for execution of therapy exercises between provider-led visits, the inclusion of an item that captures this element of patient care is consistent with recent literature that emphasizes the importance of the parents’ role in their child’s therapeutic outcomes [37, 38].

Another key thematic finding in our patients with noncomplex chronic disease was the role of the patient themselves in their own care. By focusing on parent voice, this adaptation excluded the direct voice of the child and in some cases, as explained by parent participants, made items less relevant because children managed components of their own care. Part of this was necessitated by child age and cognitive capacity of children with certain chronic health conditions. However, much of the content developed here aligns with existing and emergent literature focused on transition to adult care [39], suggesting relevance of the core constructs to these populations. Future work will include adaptation to pediatric and young adult self-report instruments for those who are cognitively capable and who take increasing responsibility for their own care.

## Limitations and future work

Despite the rigorous methods used to develop this PRO, several limitations are noted. First, the thematic analysis was based on field notes rather than transcribed interviews. This limited our ability to provide quotes in participants’ own voices. However, detailed notes included key words and rephrasing of items that parents described in the interviews, which were used to adapt item content and ensure relevance to the target population. Our sample intended to capture adolescents up to 21 but the oldest participant’s child’s age was 15 which potentially could have limited out insights in older adolescents. Additionally, we acknowledge that the sample had a limited number of father participants, which though likely a reflection of caregiver role make-up, may still have limited the measure for this participant group. Finally, this study focused on the qualitative methods for establishing face validity of the new measure and did not include field-testing of item content with a larger population to establish psychometric validation. While the measure was developed to capture nine content domains regarding parent self-efficacy, future validation of the measure will confirm whether the measure should be scored as a unidimensional measure or sub-scored by domain.

## Conclusions

The Parental Measure of Self-Efficacy Managing a Child’s Medications and Treatments is a new condition-agnostic, self-efficacy PRO measure designed for parents of children with chronic and/or disabling conditions, that is both acceptable and understandable to English and Spanish-speaking parents.

## Supplementary Information

**Additional file 1:** Form used to gather expert input on survey content.

**Additional file 2:** Form used for parental interviews.

## Data Availability

Not applicable.

## Abbreviations

ADLs: Activities of daily living
CMC: Children with medical complexity
PMCA: Pediatric Medical Complexity Algorithm
PRO: Patient-reported outcome measures
PROMIS®: Patient-Reported Outcomes Measurement Information System
SD: Standard deviation
