# Supplementary material for: Parental self-efficacy managing a child’s medications and treatments: adaptation of a PROMIS measure
Source: J Patient Rep Outcomes. 2023 Feb 3;7:10. doi: 10.1186/s41687-023-00549-z (PMC9898482; doi:10.1186/s41687-023-00549-z)
Supplement: Supplementary file 1 — Additional file 1: Form used to gather expert input on survey content. [file 41687_2023_549_MOESM1_ESM.docx]

**Additional file 1: Appendix A**

**EXPERT INPUT FORM**

**Parent Proxy Adaptation of Self-Efficacy for Medications and Treatment PROMIS Questions**

PART I. BACKGROUND AND GENERAL INSTRUCTIONS

**Project Goal**

We are adapting a survey that was written for adults with chronic medical conditions to a survey version written for parents/guardians of children with chronic medical conditions.

The original survey is written to assess an adult’s self-efficacy regarding his or her medication management and other treatment. For example in the original survey, the adult is asked to answer how confidently he/she currently feels about the following statement: “I can remember to take my medication as prescribed.” Our goal is to adapt these statements to a parent/guardian proxy voice, such as, “I can remember to give my child's medication as prescribed.”

We will also add new or different content that may be more appropriate for the care of children.

Please note that the survey is designed to be disease agnostic – meaning it would apply to most parents of children with different medical conditions, including children with medical complexity.

**Definition of Self-Efficacy**

For context, the definition of self-efficacy is an "individual’s belief in his or her capacity to perform a particular behavior or set of behaviors."

**In this case, we are interested in a parent’s *confidence in his/her abilities* to perform behaviors related to the management of his/her child’s medical condition(s)**. So this survey does not measure parents’ actual capacity to perform the behaviors.

**Your Input**

Please draw on your expertise as a care provider or parent when filling out this form. You can fill it out electronically or print it and write it on paper and send it back.

We will:

1. *First* ask you to brainstorm what sorts of behaviors parents may engage in when managing their children’s conditions.
2. *Second*, we will ask you to give feedback on the proposed adapted questions.
3. *Last*, we will ask you some short summative questions.

Our goal is that this should not take more than 1-2 hours of your time. Don’t feel like you need to dwell on any of the content, even your immediate reaction is helpful. Once we have complied your input with that of other experts, we will verify the content and wording with about 20 more parents.

**Thank you for again for your willingness to participate!**

PART II. CONTENT ELICITATION

**For this first section, we are interested in your ideas about what sorts of behaviors a parent of a child with one or more chronic conditions may need to perform in order to manage a child’s condition. Keep in mind that might be a child with asthma or a child with medical complexity. This is purposefully opened-ended and there are no “wrong” answers.**

### In the table below, please LIST any behaviors or sets of behaviors involved in managing (or caring for) a child’s medical condition(s). You don’t have to fill every line. Just write as much as you want.

### *Behaviors related to the management of a child’s medical conditions may include activity-based tasks such as giving medications but also organizational tasks like planning or having discussions with doctors. Feel free to add lines if need be buy not all lines need to be filled out. Write until you are done brainstorming.*

### After you are done listing the behaviors please think about the importance of each of these aspects of caring for a child with complex medical problems.

### On a 0-5 scale, with 0 = *Not at all important* and 5 = *Extremely important*, please rate the importance of each of these tasks, across a group of children with different medical conditions. In theory, more than one could be a 5 or a 4, though really only a few if any should be a 0.

| **List of Behaviors Involved in Caring for a Child’s Medical Conditions** | **Importance Ranking** |
| --- | --- |
| *Example: Give medication to child* | 4 |
| *Example: Participate in decisions about child’s treatment* | 5 |
|  |  |
|  |  |
|  |  |
|  |  |
|  |  |
|  |  |
|  |  |
|  |  |
|  |  |
|  |  |
|  |  |
|  |  |
|  |  |
|  |  |

PART II: REVIEW OF THE PROMIS CONTENT AND PROPOSED NEW CONTENT

**Next, we are going to ask you to review a series of item stems taken from the questionnaire.**

**In the far left column, you will read the item in the adult version. In the next column, you will see our proposed wording for the parent version. For each item, please fill out your impression of the item wording adaptation based on the 3 prompts in subsequent columns.**

***For context, the parent will be given the following instructions:***

***“****Please respond to each question or statement by marking one box per row based on your CURRENT level of confidence…”*

***☐****I am not confident at all (1),****☐*** *I am a little confident (2),* ***☐****I am somewhat confident (3),****☐*** *I am quite confident (4),* ***☐****I am very confident (5)*

| **Adult PROMIS Item for reference** | **PROPOSED Parent/Guardian Version** | **1. Was the meaning of the adapted question clear to you?**  **Yes/NO**  **If no, what was unclear?** | **2. Would you suggest any different way to word this item?**  **Yes/NO**  **If yes, what questions did you have?** | **3. Is this question relevant to your experiences with managing the care of children with chronic conditions / your child’s medical condition?**  **Yes/No**  **If no, why wasn’t it relevant?** |
| --- | --- | --- | --- | --- |
| I can take several medications on different schedules | I can give my child several medications on different schedules |  |  |  |
| I can remember to take my medication as prescribed | I can remember to give my child's medication as prescribed |  |  |  |
| I know when and how to take my medications | I know when and how to give my child's medications |  |  |  |
| I can fit my medication schedule into my daily routine | I can fit my child's medication schedule into my child's daily routine |  |  |  |
| I can follow directions when my doctor changes my medications | I can follow directions when my child's doctor(s) changes my child's medications |  |  |  |
| I can manage my medication without help | I can manage my child's medication without help |  |  |  |
| I can get help when I am not sure how to take my medicine | I can get help when I am not sure how to give my child's medicine |  |  |  |
| I can remember to refill my prescriptions before they run out | I can remember to refill my child's prescriptions before they run out |  |  |  |
| I can remember to take my medications when there is no one to remind me | I can remember to give my child's medications when there is no one to remind me |  |  |  |
| I can list my medications, including the doses and schedule | I can list my child's medications, including the doses and schedule |  |  |  |
| I can actively participate in decisions about my treatment | I can actively participate in decisions about my child's treatment |  |  |  |
| I can find information to learn more about my treatment | I can find information to learn more about my child's treatment |  |  |  |
| I can use my own judgment regarding treatment alternatives (including not having treatment) | I can use my own judgment regarding my child's treatment alternatives (including not having treatment) |  |  |  |
| I can work with my doctor to choose the treatment that seems right for me | I can work with my child's doctor(s) to choose the treatment that seems right for my child |  |  |  |
| I know what to do when my medication refill looks different than usual | I know what to do when my child's medication refill looks different than usual |  |  |  |
| I know what to do if I forget to take my medication(s) | I know what to do if I forget to give my child's medication(s) |  |  |  |
| I can use technology to help me manage my medication and treatments (for example: to get information, avoid side-effects, schedule reminders) | I can use technology to help me manage my child's medication and treatments (for example: to get information, avoid side-effects, schedule reminders) |  |  |  |
| I can continue my treatment when traveling | I can continue my child's treatment when we are traveling |  |  |  |
| I can take my medication when I am working or away from home | My child can get medication when my child is at school |  |  |  |
| I can take my medicine even if it causes mild side effects | My child can take medicine even if it causes mild side effects |  |  |  |
| I understand the difference between my symptoms and medication side effects | I understand the difference between my child's symptoms and medication side effects |  |  |  |
| I can continue my treatment when I am not feeling well | *We propose cutting this stem from child version.* |  |  |  |
| I can take my medication when there is a change in my usual day (unexpected things happen) | I can give my child's medication when there is a change in my child's usual day (unexpected things happen) |  |  |  |
| I can figure out what treatment I need when my symptoms change | I can figure out what treatment my child needs when my child's symptoms change |  |  |  |
| I can follow a full treatment plan (including medication, diet, physical activity) | I can follow my child's full treatment plan (including medication, therapy, and other care) |  |  |  |
| I can travel to my local pharmacy to fill my prescriptions | I can travel to our local pharmacy to fill my child's prescriptions |  |  |  |
| N/A – this is new content we are proposing | I can tell when my child's symptoms worsen |  |  |  |
| N/A – this is new content we are proposing | I know when my child needs to be seen by a doctor right away |  |  |  |
| N/A – this is new content we are proposing | I know what to do if my child has a medical emergency |  |  |  |
| N/A – this is new content we are proposing | I can use my child's medical equipment (safely? correctly?) |  |  |  |
| N/A – this is new content we are proposing | I can use my child's respiratory equipment (such as my child's ventilator and suction machine) |  |  |  |
| N/A – this is new content we are proposing | I can tell when my child's medical equipment's needs to be replaced |  |  |  |
| N/A – this is new content we are proposing | I know how to follow my child's feeding (or diet) plan |  |  |  |

# Summary Questions:

# Please take a moment to look over the written questions again. Do these questions, in your opinion, capture the experience of a parent caring for a child’s medical condition(s)?

___ No

___ Yes

# If No 🡪 Why not?

# Are there any other important questions we didn’t ask?

# Were there any questions in this section that seemed redundant or duplicative, meaning you think they asked such similar information that we should only ask one of them?

___ No

___ Yes

If yes 🡪 Which questions? Why?

# Is there anything else that you would like to suggest that would help us to improve these questions for future use?

**Thank you again for your time!**

**We look forward to using these questions to understand how we can better support parents and families in the care of their children.**
