# Supplementary material for: Parental self-efficacy managing a child’s medications and treatments: adaptation of a PROMIS measure
Source: J Patient Rep Outcomes. 2023 Feb 3;7:10. doi: 10.1186/s41687-023-00549-z (PMC9898482; doi:10.1186/s41687-023-00549-z)
Supplement: Supplementary file 2 — Additional file 2: Form used for parental interviews. [file 41687_2023_549_MOESM2_ESM.docx]

**Additional file 2: Appendix B**

**PARENT COGNITIVE & CONCEPT ELICITATION INTERVIEW**

Proxy Voice Adaptation of Self-Efficacy for Medications and Treatment

PART I. BACKGROUND AND GENERAL INSTRUCTIONS

**Interview Purpose**

We want to understand how to help parents/guardians care for their children with chronic medical conditions at home. Research shows that asking about how confident a patient is with their own medical care at home is a good way to keep track of how adult patients are doing. This research has not been done as much in children with complex medical conditions. That’s why we are doing these interviews.

We want your input, based on your experience as a parent, on whether the questions make sense to you, and whether you think we should ask different questions.

**What to Expect During the Interview**

We will ask you questions about your experience caring for your child and also ask you to review questions and tell us what you think of their wording.

We are audio taping this interview. The purpose of taping is to make sure we have a complete record of the discussion. Once we’ve written up the interview, we will destroy the recording. I want to assure you that no person’s name will appear in the report, and we will not include any information that would let a reader identify you specifically.

Here are some “ground rules” for our discussion:

- There is no right or wrong answer. If you are not entirely sure about the answer to a question, that is okay.
- We have a limited time. Please don’t be offended if I step in to move us ahead.

**Thank you again for participating!**

PART II. DEMOGRAPHIC QUESTIONS

**Before we begin, we would like to ask you a few questions about your background so we can compare your feedback to families with similar and different backgrounds. If you have any questions or want clarification as you go through, please let me know.**

1. Indicate participant’s gender:
   - Male (1) 🞎 Female (2)  🞎 Other/Non-Binary (3)
2. What is your age? ____ years
3. Do you consider yourself to be of Spanish /Hispanic /Latino Origin?
   - No (0) 🞎 Yes (1)
4. What is your race? (*Select as many as apply*)
   - White (1)
   - Black or African American (2)
   - American Indian/Alaska Native (3)
   - Asian (4)
   - Native Hawaiian or Other Pacific Islander (5)
   - Other (please specify ) (6)
5. What is your current marital status?
   - Never married (1) 🞎 SSeparated (4)
   - Married (2) 🞎 Divorced (5)
   - In a committed relationship (3) 🞎 Widowed (6)
6. What is the highest level of education you completed?
   - Less than high school grad/GED (1)
   - High school grad/GED (2)
   - Some college/technical degree/AA (3)
   - College degree (BA/BS) (4)
   - Advanced degree (MA, MS, MBA, PhD., MD, JD) (5)
7. What is your current employment status?
   - Full-time employed (1) 🞎 Unemployed (5)
   - Part-time employed (2) 🞎 Retired (6)
   - Full-time student only (3) 🞎 Other *______ please specify* (7)
   - Homemaker/Stay-at-home parent (4)

PART III. SELF-EFFICACY OF CHILD’S CARE CONTENT

**For the next section, we are interested in knowing about what is involved in caring for your child’s medical condition(s). We want to make sure we know everything that goes into making sure your child’s medical condition(s) is being cared for.**

**This management of your child’s medical conditions may include activity-based tasks such as giving medications but also organizational tasks like planning or having discussions with doctors.**

### Please LIST anything and everything that relates to caring for or managing your child’s medical condition(s).

### *(Interviewer—probe as needed to ensure that the parent provides a clear description of each task or behavior. Write it down in list).*

| **List of Behaviors or Tasks Involved in Caring for my Child** | **Importance Ranking** |
| --- | --- |
| *Example: Give medication to my child* | 8 |
|  |  |
|  |  |
|  |  |
|  |  |
|  |  |
|  |  |
|  |  |
|  |  |
|  |  |
|  |  |
|  |  |
|  |  |
|  |  |
|  |  |
|  |  |

### Next, I would like you to think about the importance of each of these aspects of caring for your child.

### On a 0-10 scale, with 0 = *Not at all important* and 10 = *Extremely important*, please rate the importance of each of these tasks to your child’s condition. *(Read each of the concerns listed under Question 1 and ask parent to rank it from 0-10. Indicate their ranking next to the items above*).

PART IV: REVIEW OF THE PROMIS PROXY CONTENT AND PROPOSED NEW CONTENT

**Next, I’m going to ask you to answer a series of survey questions.**

*Parent completes the drafted proxy interview questions. Make notes of any confusion or clarifications that come up when taking the questions.*

**You just completed a questionnaire called the PROMIS Self-Efficacy for Managing Medications and Treatments – Parent Proxy Version. We would appreciate your feedback regarding this questionnaire in order to determine which questions are the most appropriate to be asking parents/guardian’s of children with chronic conditions and medical complexity.**

**First, I’d like to ask you some general questions about the questionnaire.**

1. Were the instructions clear to you? ___ No (0)

___ Yes (1)

If no🡪 What was unclear about the instruction?

1. These questions ask you to respond using I am not at all confident, I am a little confident, I am somewhat confident, I am quite confident, and I am very confident.

**2a.** Did those response options make sense to you? ___ No (0)

___ Yes (1)

If no 🡪 why not?

**2b.** Was it easy to respond using those options? ___ No (0)

___ Yes (1)

If no 🡪 why not?

**2c.** Did this format provide enough response choices? ___ No (0)

___ Yes (1)

**2d.** Did this format provide too many response choices? ___ No (0)

___ Yes (1)

1. Would you say the length of the questionnaire was: ___ Too short (1)

___ Too long (2)

___ Just about right (3)

**Now we are going to go back to each item and ask some follow up questions.**

**This part can feel repetitive, but your input is important on each question item.**

Next, I’m going to ask you a series of questions about each of the items on the questionnaire.

| **Item** | **What kinds of things did you think about when you answered the question? (i.e. How did you come to the answer you gave)?** | **How would you state the question in your own words?**  (If patient struggles, remind them that it’s ok if they would say it the same way as it’s written). | **How confident were you when responding to this question?**  **1) Very Confident**  **2) Confident**  **3) Not at all confident** | **Is this question relevant to your experiences with your child’s health condition(s)?**  **Yes/No**  **If no, why wasn’t it relevant?** |
| --- | --- | --- | --- | --- |
| I know the healthcare condition(s) that affect my child |  |  |  |  |
| Etc… |  |  |  |  |
|  |  |  |  |  |
|  |  |  |  |  |
|  |  |  |  |  |

PART V. SUMMARY QUESTIONS ABOUT COGNITION

# Please take a moment to look over the questions again. Do these questions, in your opinion, capture your experience with caring for your child’s medical condition(s)?

___ No (0)

___ Yes (1)

# If No 🡪 Why not?

# Are there any other important questions we didn’t ask you?

___ No (0)

___ Yes (1)

# If Yes 🡪 Which questions? Why?

# Were there any questions in this section that seemed redundant or duplicative, meaning you think they asked such similar information that we should only ask one of them?

___No (0)

___Yes (1)

If yes 🡪 Which questions? Why?

**We have now concluded the interview.**

**Thank you for your time!**

**We look forward to using these questions to understand how we can better support parents and families in the care of their children.**
